# Supplementary material for: Transcriptomic analysis and novel insights into lens fibre cell differentiation regulated by Gata3
Source: Open Biol. 2019 Dec 18;9(12):190220. doi: 10.1098/rsob.190220 (PMC6936257; doi:10.1098/rsob.190220)
Supplement: Supplementary Material [file rsob190220supp1.pdf]

Supplementary Material to the article

Transcriptomic analysis and novel insights into lens fiber cell differentiation regulated by Gata3

Elena Martynova[1], Yilin Zhao[1], Qing Xie[1], Deyou Zheng[2], Ales Cvekl[1*]

[1]Departments of Ophthalmology and Visual Sciences and Genetics, Albert Einstein College of Medicine, Bronx, New York 10461

[2]Departments of Genetics, Neurology, and Neuroscience, Albert Einstein College of Medicine, Bronx, New York 10461

* To whom correspondence should be addressed: Albert Einstein College of Medicine, 1300 Morris Park Ave., Bronx, NY 10461. Tel.: +1-718-430-3217; E-mail: ales.cvekl@einstein.yu.edu.

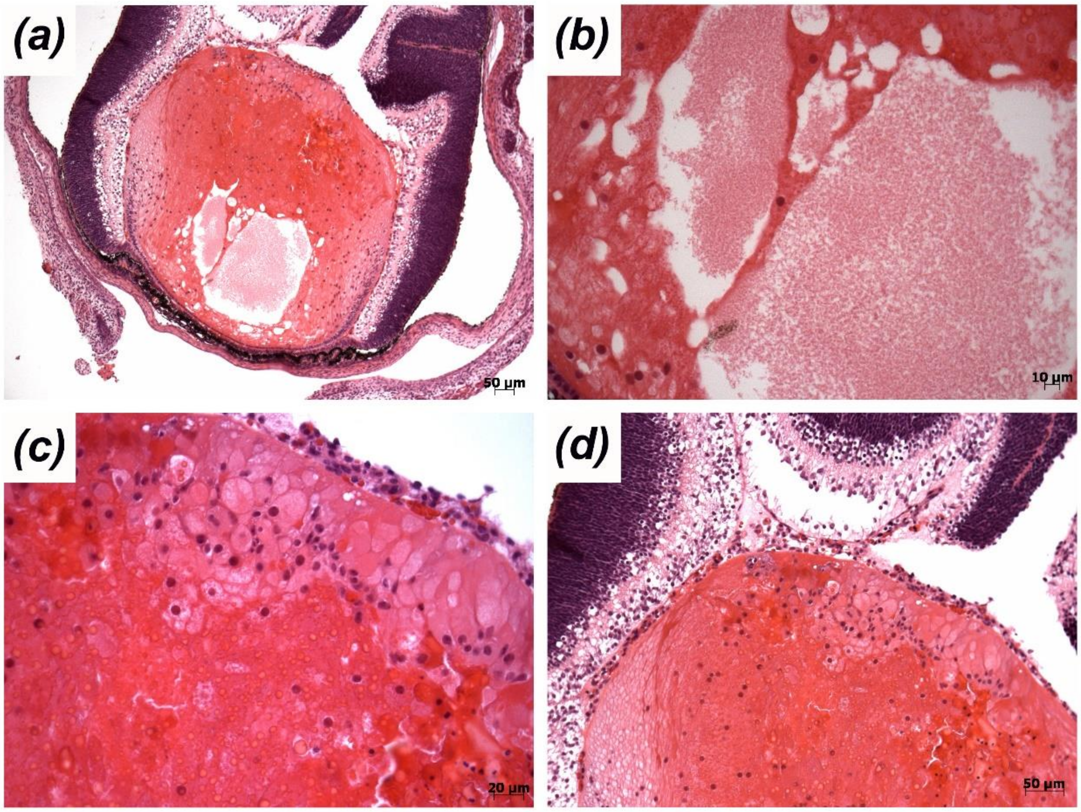

Figure S1

Higher magnification for visualization of abnormal lens fiber cell differentiation and retention of nuclei. (A) Whole lens, (B), (C), (D), higher magnifications. Scale bars: A, 50 µm; B, 10 µm; C, 20 µm; D, 50 µm.

(a)

Sample	Total reads	After deDup	Trim galore	Align (concordant pairs)
KO-1	21651177	48%	21619299	83.8%
KO-2	26897887	47%	26860470	83.8%
KO-3	25690341	48%	25655182	84.1%
WT-1	22445181	46%	22415023	83.7%
WT-2	22899585	42%	22860963	83.7%
WT-3	24549238	49%	24519758	84.1%

(b)

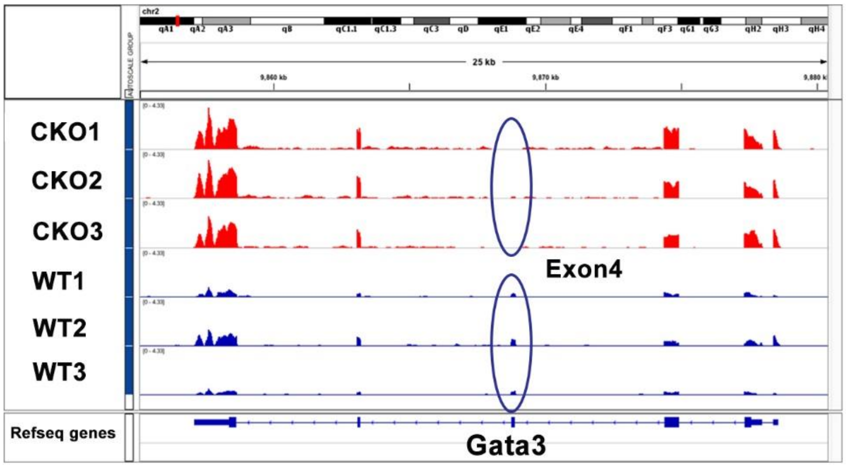

Figure S2

RNA-Seq data of Gata3 mutated lenses. (A) Statistical summary of RNA-seq data. (B) Confirmation of the deletion of Gata3 exon 4.

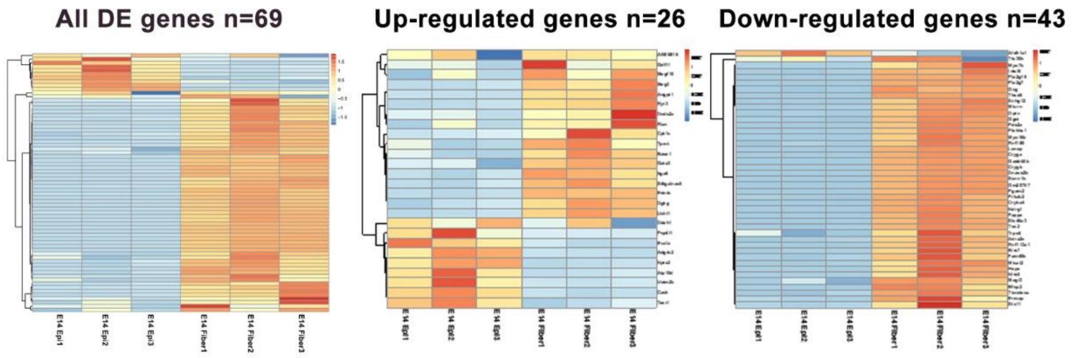

Figure S3

The heatmaps of normalized gene expression levels for differentially expressed genes in Gata3 mutated lenses at E14.5 epithelium and fiber. Three up regulated genes in Gata3 mutated lenses were not detected in E14.5 epithelium or fiber, including 4930412O13Rik, Eda2r, and Sstr1.

(a)

Gata3 DE gene promoters enriched top motifs

DE gene list	Top enriched motif (JASPAR)	Motif name	q-value	% of Target Sequences with Motif	% of background Sequences with Motif
All DE genes	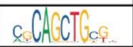	HLH-1	0.0012	89.25	76.02
Up-regulated	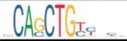	MyoD	0.0004	96.05	75.25
Down-regulated	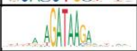	GATA	0.0001	41.07	20.30

Gata3 motif containing genes:

Up: Gdf11, Cpt1c, Tpmt, Gata3, 4930412O13Rik

Down: Pgam2, Dnase2b, Pde2a, Scnn1b, Optn, Ggct, Pla2g16, Rnf180, Tsc2, Thsd4, Plekhs1, Cryga, Fam46b, Slc46a3, Gcg, Crygb, Timeless

(b)

Gsea deseq2 log2fc

Upregulated Nominal p value<0.01

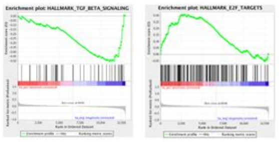

Downregulated

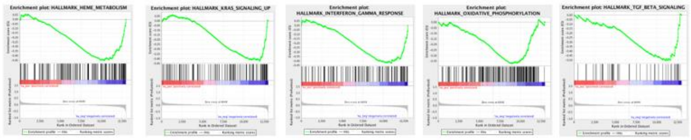

Figure S4

Functional analysis of Gata3-regulated genes. A. Prediction of enriched motifs at the promoters of Gata3-regulated genes using Homer. The top motifs were listed and then scanned against Gata3-regulated genes to find the genes with Gata3 motifs. B. Enriched functions of up and down regulated genes in Gata3 mutated lenses using GSEA (p<0.01).

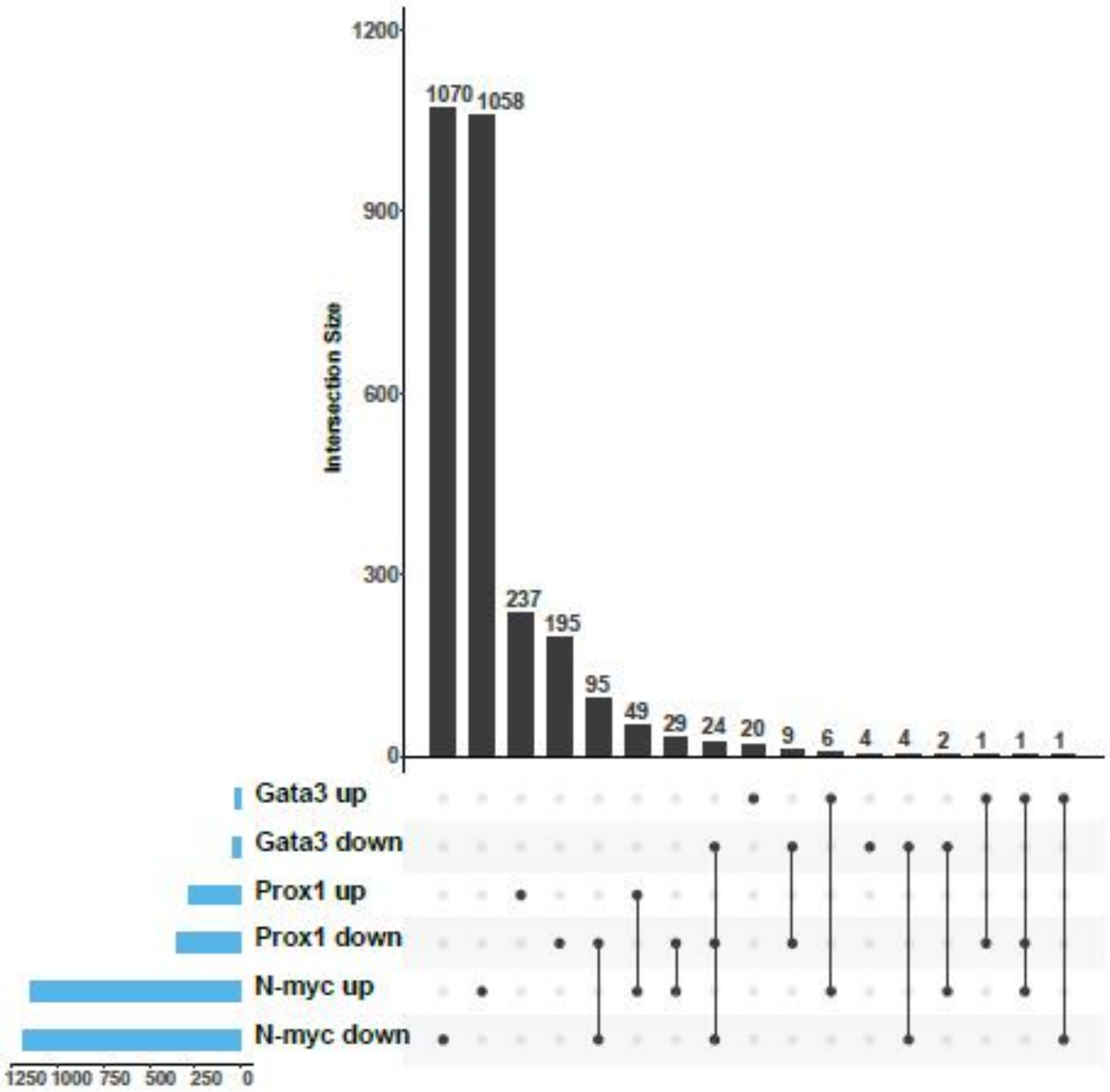

Figure S5

Histograms of shared and unique differentially expressed genes in Gata3, N-Myc and Prox1 CKO lenses. The horizontal bars (and numbers) represent the numbers of up/down regulated genes comparing KO to WT in each study. The vertical bars indicate how many of those genes are shared or unique to different comparisons as marked by linked dots.
